# Improving the diagnosis and treatment of congenital heart disease through the combination of three-dimensional echocardiography and image guided surgery

**DOI:** 10.1186/s12880-024-01235-2

**Published:** 2024-03-13

**Authors:** Yong Jiang

**Affiliations:** ZhongShan Hospital of Xiamen University, School of Medicine Xiamen University, Xiamen, Fujian 361004 China

**Keywords:** Congenital heart disease, Three-dimensional echocardiography, Image guided surgery, Real-time imaging, Two-dimensional ultrasound

## Abstract

**Objective:**

The paper aimed to improve the accuracy limitations of traditional two-dimensional ultrasound and surgical procedures in the diagnosis and management of congenital heart disease (chd), and to improve the diagnostic and therapeutic level of chd.

**Method:**

This article first collected patient data through real-time imaging and body surface probes, and then diagnosed 150 patients using three-dimensional echocardiography. In order to verify the effectiveness of the combination therapy, 60 confirmed patients were divided into a control group and an experimental group. The control group received conventional two-dimensional ultrasound and surgical treatment, while the experimental group received three-dimensional ultrasound and image guided surgical treatment.

**Result:**

In the second diagnosis, the diagnostic accuracy of type 1, type 2, and type 3 in the control group was 84.21%, 84.02%, and 83.38%, respectively. The diagnostic accuracy rates of type 1, type 2, and type 3 in the experimental group were 92.73%, 92.82%, and 92.83%, respectively. In the control group, 2 males and 1 female experienced heart failure after surgery. However, in the experimental group, 0 males and 0 females experienced heart failure after surgery.

**Conclusion:**

The combination of three-dimensional echocardiography and image guided surgery can improve diagnostic accuracy and surgical treatment effectiveness, thereby reducing risks and complications, and improving surgical success rate.

## Introduction

The symptoms and manifestations of CHD can vary greatly, depending on the specific location and degree of cardiac structural abnormalities in different patients. Common symptoms include shortness of breath, purple lips, and susceptibility to fatigue. In addition, CHD can have adverse effects on patients’ physical health and emotional and social abilities during their growth and development. For different types of CHD, different diagnostic and treatment methods are required. However, traditional two-dimensional ultrasound has limited image acquisition ability and unclear display of cardiac reconstruction structure. The risk of cardiac catheterization surgery is high, resulting in certain postoperative complications and seriously affecting the rehabilitation effect and quality of life of patients. The combination of three-dimensional echocardiography and image guided surgery can more accurately diagnose CHD, and improve the success rate and efficacy of surgery. It can guide doctors in real-time during surgery, shorten surgical time, and reduce surgical difficulty and the incidence of postoperative complications. At the same time, three-dimensional echocardiography and image guided surgery can also provide doctors with more accurate surgical plans, improving the success rate and safety of surgery [1].

With the development of social economy and the continuous progress of medical technology, the level of medical care and the quality of medical services are also constantly improving. However, in clinical practice, CHD remains a relatively difficult disease to treat. The purpose of Desai Kinjal was to improve the efficiency of diagnosing CHD for smooth treatment. In recent years, the newly implemented pulse oximetry screening for severe CHD improved the diagnostic rate of heart disease. However, many sick infants often lost compensation before being screened in neonatal nurseries, as delayed diagnosis leads to worsening of the patient’s condition [2]. Arnaout Rima found that CHD was a common congenital defect. Fetal ultrasound screening provided cardiac images, and cardiac measurements were related to reported normal and abnormal cardiac measurements. The ensemble learning model could significantly improve the detection of fetal coronary heart disease, which was a key and global diagnostic problem [3]. Prenatal routine obstetric ultrasound examination for the diagnosis of complex CHD could improve postpartum prognosis, but its sensitivity was low. Letourneau Karen M. reported a project aimed at improving prenatal detection of complex CHD by implementing a specific routine prenatal screening program, and found that implementing this program coould significantly increase the detection rate of critical heart abnormalities [4]. To achieve early diagnosis and precise treatment of CHD, advanced medical technology and equipment are needed to provide safer and more effective diagnosis and treatment services for patients.

With the advancement of surgical and diagnostic techniques, the incidence of CHD in adults continues to increase. It is important for doctors to use non-invasive imaging techniques to supplement examinations and evaluate patients. Although some patients have regular cardiac follow-up, some may be new patients and may not even know their cardiac history. Mcleod George highlighted some advances in the diagnosis of CHD, including three-dimensional echocardiography, which was proved to be a useful tool, and its utility rapidly expanded with the development of better and new technologies [5]. Di Salvo Giovanni found that progress was made in the diagnosis and treatment of CHD, and the survival rate of adult patients with CHD significantly improved. However, patients with CHD were a heterogeneous population, and residual anatomical and hemodynamic abnormalities were very common. With the continuous aging of the population with CHD, efficient cardiac imaging evaluation was needed [6]. Medvedofsky Diego suggested that three-dimensional echocardiography provided an opportunity to accurately quantify the shape of the heart. He retrospectively studied hospitalized patients referred by transthoracic echocardiography. These patients had high-quality three-dimensional echocardiography images [7]. Chinh Nguyen Dinh proposed a signal processing method that combines empirical mode decomposition and continuous wavelet transform for short-term estimation of heart rate and beat time from radar signals. We evaluated the performance of the proposed method using 85 dengue fever patients and 40 healthy subjects. Subsequently, the estimated non-contact HR will be compared with the non-contact HR of commercial contact medical devices. The results indicate that heart rate can be estimated within 5 s with an accuracy of 96.2 ± 2.5% [8]. Hui Tran Quang proposed a distorted Born iterative method based on first-order Born approximation, which was used for diffraction tomography imaging to obtain many measurement values from the number of transmitters and receivers. Given that biomedical images are usually sparse, compressed sensing technology can effectively apply to ultrasound tomography by reducing the number of transmitters and receivers while maintaining high-quality image reconstruction [9]. CHD is a kind of congenital malformation with high incidence, and its incidence rate is quite high. Therefore, it has important clinical significance for the early diagnosis and treatment of CHD.

CHD is a common cardiovascular disease in infants and children, and traditional diagnosis and treatment methods have certain limitations. This article aimed to explore the combined use of three-dimensional echocardiography and image guided surgery to improve the diagnosis and treatment of CHD. The combination of three-dimensional echocardiography and image guided surgery has become an important method for the diagnosis and treatment of CHD. Image guided surgery has the advantages of precision, non-invasive, and non-thoracotomy, which can improve the effectiveness of surgery and improve the survival rate and quality of life of patients. With the continuous development of medical technology, three-dimensional echocardiography and image guided surgery have become important means for the diagnosis and treatment of CHD. Strengthening the research and application of three-dimensional echocardiography and image guided surgery would help improve the diagnosis and treatment level of CHD, thus bring better treatment experience and effectiveness to patients.

## Materials and methods

Three-dimensional echocardiography is a non-invasive examination technique with advantages such as high resolution, high sensitivity, high specificity, and no radiation. It can obtain fine images of the heart of CHD patients without the use of radioactive substances [10, 11]. At the same time, three-dimensional echocardiography can also display real-time dynamic changes within the heart, providing accurate guidance for image guided surgery [12].

### Application of three-dimensional echocardiography in the diagnosis of CHD

#### Principles of three-dimensional echocardiography technology

**Fig. 1:**
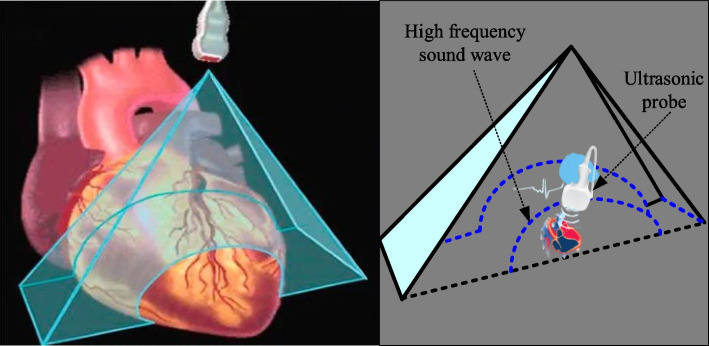
Principle of three-dimensional echocardiography

Three dimensional echocardiography refers to the collection of a series of two-dimensional ultrasound images inside the heart and reconstruction of them into three-dimensional images through a computer. The implementation of this technology relies on the physical characteristics of ultrasound, which is a high-frequency sound wave with a frequency much higher than that heard by the human ear. The principle of three-dimensional echocardiography is shown in Fig. 1:

As shown in Fig. 1, the principle of ultrasound generation inside the heart is to use high-frequency sound waves detected by ultrasound to reflect and absorb different tissues within the heart.

Three-dimensional echocardiography is a non-invasive cardiac examination technique that obtains data through real-time imaging, body surface probes, and other methods. It can use ultrasound imaging principles to obtain three-dimensional images of the human heart using body surface probes. Its principle is to combine multiple two-dimensional images into one three-dimensional image, in order to observe the structure and function of the heart more clearly. Three-dimensional echocardiography can help doctors diagnose more accurately and provide better treatment plans. Compared with two-dimensional ultrasound imaging, three-dimensional echocardiography provides an additional one-dimensional space on the basis of two-dimensional imaging. The reconstructed image provides much more information and resolution for fine structures than two-dimensional methods, making it easier for ultrasound workers and clinical doctors to understand and accept, improving the accuracy and reliability of ultrasound diagnosis. Three dimensional echocardiography has a dynamic display function, especially in the field of cardiovascular disease. It is easy to clarify the three-dimensional shape, lesion range, and the relationship between adjacent structures of cardiovascular diseases under dynamic conditions, and has great application value in the diagnosis and treatment of diseases. Three dimensional echocardiography can directly display the three-dimensional shape of the heart cavity without making any geometric assumptions about the ventricular cavity. It is also not affected by the irregular geometric shape of the heart cavity during the lesion, so it is more accurate than the two-dimensional ultrasound measurement method. The three-dimensional capacity measurement method is the three-dimensional volume method. After the three-dimensional database is built, the display mode of the image is determined, the reference section of the image is adjusted, and then the selected area of interest is extracted.

The walls of the coronary arteries in the human body are divided into three layers: intima, media, and adventitia. At birth, each person's blood vessels are unobstructed and smooth. With the growth of age, especially with multiple risk factors such as hypertension, diabetes and smoking, endothelial cells, the "barrier" of our vascular wall, are damaged. The "bad" cholesterol (low-density lipoprotein cholesterol, LDL-C) in the blood, after being oxidized, penetrates through the damaged "barrier" into the vascular intima and gradually deposits, forming "atherosclerotic plaque". The accumulation of plaque causes the narrowing of the lumen. Pathological studies show that the process of atherosclerosis has begun in adolescence. Therefore, this article selected 150 suspected and confirmed CHD patients (3–6 years old) from a certain people’s hospital for data collection, and obtained patient data through real-time imaging and body surface probes. Finally, the diagnosis was made.

##### Real time imaging

Real time imaging is the most commonly used acquisition method for three-dimensional echocardiography, which constructs three-dimensional images by synthesizing multiple two-dimensional images. The data reconstruction process is as follows: first, the camera pose of each perspective is restored from photos from multiple perspectives, and the sparse structure of the scene is estimated to obtain the depth map of each perspective, thereby obtaining a single perspective point cloud. Then point clouds from various perspectives can be fused and surface reconstruction can be performed. This process is called data reconstruction, and the time resolution of real-time imaging is relatively high. Multiple images can be obtained in a short time and synthesized into a three-dimensional image. The reconstruction formula is as follows:

1 $$V = \sum\nolimits_{i = 1} {A_{i} }$$

Multiple ultrasound probes $A_{i}$ are used in real-time imaging, which can rotate or move in different directions to observe the heart from multiple angles. This method can provide more data for doctors to analyze the structure and function of the heart more comprehensively.

The imaging formula for three-dimensional echocardiography is as follows:

2 $$I\left( {x,y,z} \right) = \int {\left( {x^{'} ,y{^{'}} ,z{^{'}} } \right)} G\left( {x - x{^{'}} ,y - y{^{'}} ,z - z{\prime} } \right)dx{^{'}} dy{^{'}} dz{^{'}}$$

Among them, $\left( {x^{'} ,y^{'} ,z^{'} } \right)$ is the ultrasound signal emitted by the probe, and $G\left( {x - x^{'} ,y - y^{'} ,z - z^{'} } \right)$ is the reflection signal of the ultrasound after passing through the heart tissue.

Based on the heart movement speed and time of 150 patients, the position of the heart at a certain time point is calculated. By collecting multiple two-dimensional images at different time points and calculating the position of the heart, a three-dimensional image of the heart can be constructed. The position calculation is as follows:

3 $$A = ht$$

Among them, $A$ represents the position of the heart; $h$ represents the movement speed of the heart; $t$ represents time.

**Fig. 2:**
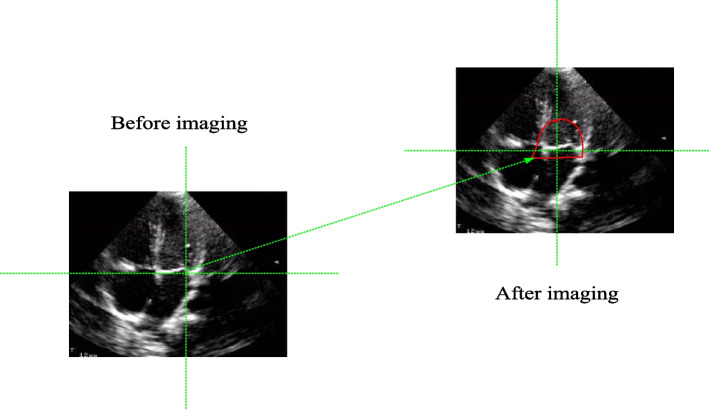
Schematic diagram of real-time imaging

Through real-time imaging, doctors can observe the heart in real-time and analyze and diagnose it. Real time imaging can obtain images of abnormal parts in a short period of time, as shown in Fig. 2:

As shown in Fig. 2: Before imaging, it is not possible to see the specific areas with heart problems. After imaging, the problematic areas are circled in red.

Real time imaging also has high spatial resolution, which can display the subtle structure of the heart, effectively assisting doctors in diagnosis and treatment. Real time imaging has important value in clinical applications. Compared with traditional two-dimensional ultrasound, real-time imaging can provide more comprehensive and accurate cardiac information for CHD patients, providing better diagnostic basis for doctors. With the continuous development and innovation of technology, the application prospects of real-time imaging would be more broad [13, 14].

##### Body surface probe

Body surface probes are another commonly used method for three-dimensional echocardiography, in which ultrasound can be transmitted to the heart through the body surface to obtain three-dimensional images. Compared with real-time imaging, body surface probes are suitable for examining different parts of the heart. In addition, body surface probes can simultaneously obtain images from multiple sections, which helps doctors diagnose and evaluate lesions more accurately [15].

Placement of probe: the probe is placed on the chest of 150 patients, and the position and angle of the probe need to be adjusted according to the patient’s condition.

Starting scanning: the probe contacts the heart through the skin, emits ultrasound, and receives echo signals.

The propagation distance of ultrasonic signals $d_{v}$:

4 $$d_{v} = \frac{{s_{v} \times t_{i} }}{2}$$

Among them, sound speed $s_{v}$ is a known constant, and $t_{i}$ is the return time of ultrasound. After being emitted from the probe, ultrasound propagates through the heart tissue and is reflected by the tissue before being received by the probe. The return time is proportional to the distance traveled by ultrasound in the tissue.

The scanned data is collected and processed, and high-frequency sound waves are emitted into the patient’s chest through the probe, which are reflected back through the tissue. The ultrasound machine receives these echo signals and converts them into digital signals, forming a series of data.

The calculation formula for cardiac function indicators in three-dimensional echocardiography is as follows:

5 $$W_{q} = E_{q} - E_{s}$$

Among them, $W_{q}$ represents the stroke output; $E_{q}$ represents end-diastolic volume; $E_{s}$ represents the end systolic volume.

The formula for calculating cardiac contractility in three-dimensional echocardiography is as follows:

6 $$E_{f} = \frac{{W_{q} }}{{E_{q} }}$$

$E_{f}$ represents the contractile force of the heart. Three-dimensional echocardiography is a non-invasive, safe, convenient, and accurate cardiac examination method that can obtain high-quality three-dimensional images of the heart in a short period of time and evaluate the structure and function of the heart.

In a quiet state, normal individuals have a stroke output range of 60–80 ml (ml), a normal left ventricular end diastolic volume of 75-160 ml, and a systolic end systolic volume of approximately 75 ml. However, patients with CHD cannot reach normal values. In order to reduce the differences in the collection process, two repeated collection processes were conducted, and each collection was based on the average value of the patient. The data obtained from the two collections are shown in Table 1:

**Table 1:** Situation of 150 undiagnosed patients

Index	1	2
Stroke volume(ml)	55.31	55.28
End-diastolic volume(ml)	60.42	59.93
End-systolic volume(ml)	61.25	61.17

As shown in Table 1: In the first collection, the average stroke output of 150 undiagnosed patients was 55.31 ml. The average end diastolic volume was 60.42 ml, and the average end systolic volume was 61.25 ml.

##### Image reconstruction

The collected data is processed and calculated using mathematical models, such as temporal image reconstruction methods. The discrete Fourier transform describes the conversion process from spatial domain to frequency domain. By performing the discrete Fourier transform on the pixel values of the original image, spectral information in the spatial domain can be obtained.

The discrete Fourier transform formula is as follows:

7 $$F\left( {u,v,w} \right) = \sum {f\left( {a,b,z_{i} } \right)} * \frac{{b_{n} }}{N}$$

Among them, $f\left( {a,b,z_{i} } \right)$ represents the pixel value of the original image.

Image reconstruction can generate a three-dimensional image of the heart from the collected data. The discrete Fourier transform is based on imaging principles and achieves the conversion from two-dimensional data to three-dimensional images by transforming and processing the original image pixels [16]. The generated three-dimensional images can be displayed and analyzed through computer software, and doctors can observe the three-dimensional structure of the heart, blood flow dynamics, and other information on the screen for diagnosis and evaluation.

After image reconstruction, patients who have been diagnosed and those who have not yet been diagnosed (not CHD) would be distinguished, as shown in Table 2:

**Table 2:** Patient situation after diagnosis

Basic information	Index	Number of people	Proportion (%)
Diagnosis status	make a definite diagnosis	102	68
	Undiagnosed	48	32
Gender (make a definite diagnosis)	Male	56	55
	Female	46	45
Age(make a definite diagnosis)	3–4 years old	51	50
	5-6 years old	51	50

As shown in Table 2, the number of confirmed cases was 102, accounting for 68%, while the number of undiagnosed cases was 48, accounting for 32%; there were 56 confirmed cases of male gender and 46 confirmed cases of female gender.

#### Application of three-dimensional echocardiography in the diagnosis of CHD

For complex CHD, traditional two-dimensional echocardiography often cannot comprehensively evaluate the complex structure of the heart, while three-dimensional echocardiography can provide more accurate anatomical and functional information of the heart, helping doctors better understand and diagnose these complex lesions [17, 18].

##### Application in valve defects

Three-dimensional echocardiography can provide detailed anatomical structure and functional information of the valve. For the diagnosis of valve defects, it can accurately evaluate the opening and closing status, degree of reflux, and other pathological features of the valve, helping doctors determine whether surgical repair or replacement is necessary [19]. By observing three-dimensional images, doctors can accurately evaluate important parameters such as valve shape, size, and position, which is crucial for determining the type and severity of valve defects. For mitral regurgitation, the severity of regurgitation can be observed during mitral valve closure. Based on the information provided by three-dimensional echocardiography, doctors can accurately evaluate the pathological characteristics and functional status of the valve, helping to determine whether surgical repair or replacement is necessary.

Compared with traditional two-dimensional ultrasound, real-time three-dimensional echocardiography can intuitively display the three-dimensional morphology of the mitral and tricuspid valves, and can display the cross-sectional orientation of the heart structure in the form of a bird’s-eye view. It can directly observe the complete morphology and real-time activity of the mitral and tricuspid valve leaflets. Real time three-dimensional echocardiography is clearer in distinguishing the number and morphology of valve leaflets [20]. Real time three-dimensional echocardiography can clearly display the morphology and subvalvular devices of each valve, and clarify the boundaries and accurate valve area of the valve. In the diagnosis and treatment of some CHD such as congenital valve malformations, real-time three-dimensional echocardiography can accurately present the shape, location, severity, and cumulative range of the lesion, providing more favorable basis for clinical diagnosis and treatment [21].

##### Application in atrial septal defect

Three dimensional echocardiography plays an important role in the diagnosis and evaluation of congenital heart ward septal defects. Atrial septal defect is a simple CHD. It refers to a defect in the interventricular septum (the wall separating the left and right ventricles) of the heart, resulting in channels or gaps between the left and right sides of the heart. This situation may lead to a mixture of oxygen enriched and oxygen anemia blood, as oxygen enriched blood usually comes from the left ventricle, while oxygen anemia blood comes from the right ventricle. And the current treatment methods are relatively mature. Generally, interventional closure or repair surgery can be used to cure it. Conventional two-dimensional ultrasound basically meets the diagnostic criteria for the morphology and structure of atrial septal defects, and two-dimensional ultrasound cannot directly display the defect status. Therefore, real-time three-dimensional echocardiography can be used to directly observe the overall shape of atrial septal defects and the relationship between surrounding tissue structures through the left and right atria.

Three-dimensional echocardiography can evaluate the size, location, and morphology of atrioventricular septal defects. By observing the heart, the type of defect can be determined, helping doctors develop appropriate treatment plans, including surgical repair or transcatheter closure.

**Fig. 3:**
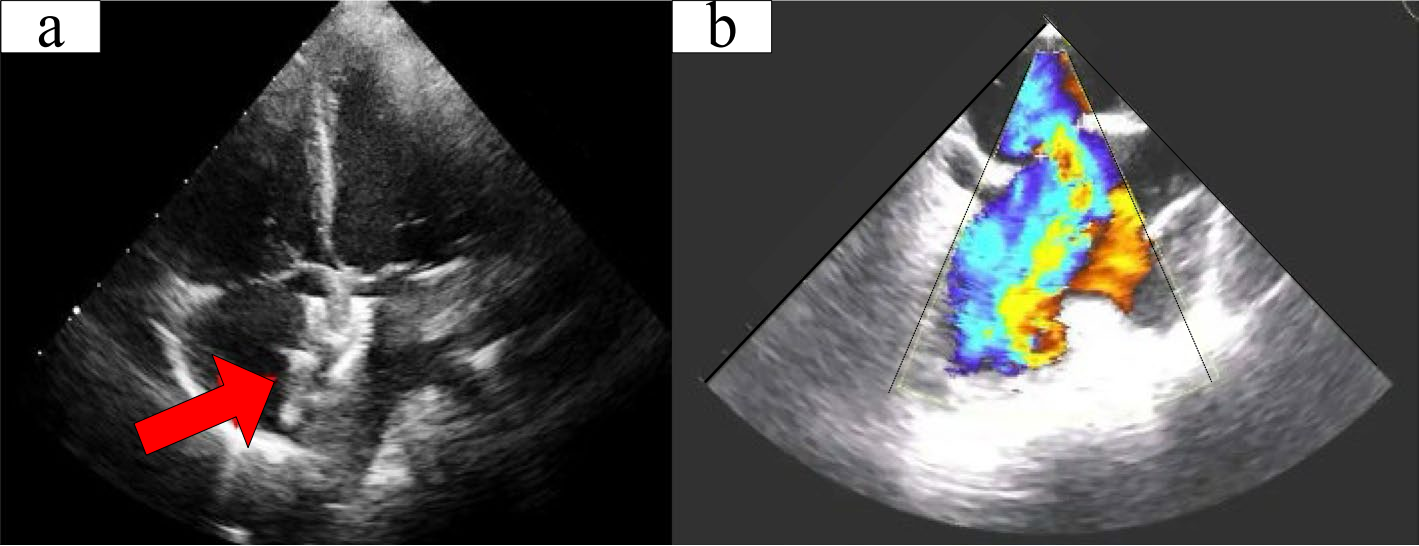
Comparison between two-dimensional ultrasound and three-dimensional echocardiography

The comparison between two-dimensional ultrasound and three-dimensional echocardiography is shown in Fig. 3:

As shown in Fig. 3, the two-dimensional ultrasound image (a in Fig. 3) is not very clear and only the red arrow indicates the approximate location. However, through the three-dimensional echocardiography (b in Fig. 3), doctors can obtain a true and clear heart image for accurate positioning.

Three-dimensional echocardiography helps to determine the specific location of the defect, including whether it is located in the central or marginal area of the ventricular septum, as well as measuring the diameter and area of the defect. Three-dimensional echocardiography can observe the flow of blood in the heart, and doctors can evaluate the degree and direction of blood reflux from the left ventricle to the right ventricle by observing the degree and direction of reflux. This helps to assess the severity and scope of the condition.

Three-dimensional echocardiography has many advantages in the diagnosis of CHD, providing comprehensive and accurate information on cardiac structure and function. In the diagnosis of diseases such as valve defects and atrial septal defects, three-dimensional echocardiography can help doctors better understand the type and degree of lesions, formulate appropriate treatment plans, and play an important role in surgical navigation. With the continuous innovation and development of technology, the application prospects of three-dimensional echocardiography in the field of CHD would be even broader.

### Application of image guided surgery in the treatment of CHD

Image guided surgery is a precise, non-invasive, non-thoracotomy surgical technique that provides precise guidance information for surgery by accurately locating the surgical site using three-dimensional echocardiography. At the same time, image guided surgery can also improve the success rate of surgery, and improve the survival rate and quality of life of patients. Image guided surgery helps doctors accurately locate and locate anatomical structures during the surgical process by aligning and registering pre acquired medical images with actual surgical scenes, thereby improving the accuracy and safety of the surgery.

#### Principles of image guided surgery

##### Image navigation in image guided surgery

Before surgery, patients need to undergo relevant medical imaging examinations to obtain high-resolution images of specific areas of the patient, which can present detailed information such as tissue structure, lesion location, and vascular distribution. During surgery, doctors use special devices such as navigation systems to combine the aligned images with the actual surgical scene. These devices can monitor the position of surgical instruments and patients in real-time through trackers and sensors, and display the overlap between medical images and the actual surgical scene on the display screen. Image navigation is a key step in image guided surgery, which aligns and registers pre acquired medical images with actual surgical scenes, and provides real-time visual navigation and guidance. Image guided surgery can reduce the risk of tissue damage and postoperative complications, and is favored due to its smaller trauma and less interference with surrounding tissues. The personalized treatment characteristics of this technology enable doctors to perform customized surgeries based on the specific anatomical structure and condition of patients, improving the accuracy and effectiveness of the surgery. Faster postoperative recovery is also one of its advantages, as patients can usually return to normal life in a shorter period of time. 3D ultrasound and imaging technology can provide more vivid and accurate 3D physiological and pathological images of the human body, making it easier for doctors to determine the complex structures and anatomical relationships such as the level, shape, and blood vessels of lesions. At the same time, virtual surgery can provide immersive surgical scenario simulation for medical training and surgical rehearsals, helping doctors efficiently master surgical techniques, improve preoperative diagnostic efficiency, and further reduce surgical risks. Therefore, image-guided surgery is of great significance in modern medicine. It not only provides patients with safer and more accurate treatment choices, but also provides doctors with more convenience and accuracy in operation, expanding its application scope to multiple medical fields and becoming an indispensable part of the medical field.

Medical image data is preprocessed and segmented to obtain key anatomical structure and lesion area information. This information is fused with the real-time position of the surgical instruments and patients, allowing doctors to accurately understand the position, orientation, and distance of the surgical scene and related anatomical structures through the superimposed images on the display screen.

Image navigation utilizes computer science and medical imaging technology to align and register pre acquired medical images with the actual anatomical structure of the patient. This is usually achieved through image registration. Image registration plays a crucial role in image navigation. It is the process of aligning and matching medical images from different times, modalities, or sources to ensure that they accurately correspond to the actual anatomical structure of the patient in the same coordinate system. Image registration ensures that images from different times or sources are aligned in the same spatial coordinate system, enabling doctors to accurately locate and navigate surgical tools or treatment equipment. This is crucial for the accuracy and success of surgical procedures. The registered images can be used for intraoperative or postoperative monitoring and evaluation. Doctors can compare preoperative and postoperative images, observe treatment outcomes, evaluate surgical success, and assess patient recovery.

Rigid body transformation describes the process of mapping the pixel coordinates of the original image to physical coordinates. Rigid body transformation is a rigid transformation that maintains the shape and size of the object, only performing translation, rotation, and scaling. In image navigation, rigid body transformation is commonly used to align pre acquired cardiac medical images with the patient’s actual cardiac structure. The mathematical expression of rigid body transformation is as follows:

8 $$K\left( {i,j} \right) = R * P\left( {i,j} \right) + T_{0}$$

Among them, $\left( {i,j} \right)$ represents the pixel coordinates in the original cardiac medical image; $P\left( {i,j} \right)$ represents the coordinates in the corresponding physical space; $R$ represents the rotation matrix; $T_{0}$ represents the translation vector.

In image navigation, affine transformation is commonly used to accurately align and register cardiac medical images with actual cardiac structures. The mathematical expression of affine transformation $U\left( {i,j} \right)$ is as follows:

9 $$U\left( {i,j} \right) = A * P\left( {i,j} \right)^{{T_{i} }} + T_{0}$$

In medical image processing, affine transformation is used to align and register different medical images, ensuring that they accurately match the actual anatomical structure in the same coordinate system. Affine transformation can take into account changes in the translation, rotation, and scaling of the heart in different images. By performing affine transformations on different images of the heart, these images can be accurately aligned to ensure perfect overlap in the same coordinate system, providing accurate anatomical information for surgical planning and navigation. The goal of image registration is to find the optimal transformation parameters (such as rotation angle, translation distance, etc.), so that the pre acquired cardiac medical image can be aligned with the actual anatomical structure of the patient. Image registration in image navigation is a core technology that describes the process of aligning pre acquired cardiac medical images with the actual anatomical structure of patients. Image registration can help doctors diagnose heart disease more accurately, plan and operate surgeries, and improve treatment effectiveness and surgical safety.

##### Real time imaging in image guided surgery

Image guided surgery can provide real-time imaging capabilities. By using real-time imaging devices such as X-ray imaging, ultrasound, optical coherence tomography, etc., doctors can obtain real-time anatomical structure information during the surgical process. These real-time imaging technologies can help doctors observe changes in the surgical area in real time and perform accurate operations.

X-ray imaging can display the bone structure and certain soft tissue features in a patient’s body. During surgery, doctors can use X-ray machines to transmit real-time X-ray images to a display screen to observe the position and changes during the surgery process. Ultrasound imaging utilizes high-frequency sound waves to image human tissues, which has the advantages of non-invasive and strong real-time performance. During the surgical process, doctors can use ultrasound probes to transmit real-time ultrasound images to the display screen, helping doctors observe the anatomical structure and blood flow status of the surgical area.

#### Application of image guided surgery in the treatment of CHD

Image guided surgery is an important technology in the medical field. It can provide doctors with images of the lesion area for surgical planning before surgery, and can locate the spatial position information of surgical instruments in real-time during surgery. It presents doctors with three-dimensional spatial structure information of surgical instruments in the lesion area, enabling them to accurately and effectively perform surgical operations. Image guided surgery is divided into two main categories based on the time taken to obtain images. One is the guidance of preoperative image fusion, and the other is the real-time guidance of intraoperative image fusion.

##### Image navigation

In traditional surgery, traditional Chinese medicine doctors operate with the naked eye or limited intuition, but the complexity of the heart structure makes it difficult to intuitively determine the position and direction during the surgery process. Through image guided surgery, doctors can obtain high-resolution image guidance in real-time, such as stereoscopic images, navigation maps, etc., to help doctors accurately locate and operate. This real-time image guidance can greatly reduce surgical risks and improve the accuracy and success rate of surgery.

The image navigation system can align and register the pre acquired cardiac images with the actual surgical scene in real-time, allowing doctors to accurately understand the position, orientation, and distance of the surgical scene and related anatomical structures through the superimposed images on the display screen. For complex CHD surgeries, doctors can use image navigation systems to guide the navigation and positioning of surgical instruments, ensuring the accuracy and safety of surgical operations. Doctors can accurately understand the patient’s heart structure, lesion location, blood vessel routing, and other information through image navigation systems, thus making preoperative planning. This helps doctors determine the optimal surgical path and strategy, assess surgical risks in advance, and prepare for surgery. Before surgery, the system will register and align medical images from different times or sources to ensure accurate matching in the same coordinate system. These registered images provide detailed information on the surgical path and target structure. During surgery, the system can track surgical tools, patient body parts, and predetermined surgical paths in real-time. By combining real-time image data and pre planned paths, the system can provide real-time navigation information to help doctors accurately locate and operate.

##### Operating instructions

The image navigation system can provide real-time visual navigation and guidance, helping doctors perform surgeries according to predetermined paths and targets. This helps doctors follow the predetermined surgical plan, reduce operational risks, and improve the accuracy and success rate of the surgery.

During the surgery process, doctors use an image navigation system for real-time navigation and positioning. The system would register real-time X-ray, ultrasound, or other imaging technology images with pre-operative planned models and display them on the operating room display screen. Through real-time navigation and positioning, doctors can avoid cutting or damaging normal tissues, and perform surgical operations more accurately. The image navigation system can provide visual navigation of surgical instruments, helping doctors accurately guide surgical instruments. On the screen, doctors can see the position, direction, and motion trajectory of surgical instruments. This enables doctors to better control surgical instruments, accurately enter the lesion area, and complete corresponding repairs or treatments.

## Results

### Selection criteria

The optimal age for coronary heart disease surgery is between 3 and 6 years old, with 60 out of 102 confirmed coronary heart disease patients selected.

Inclusion criteria: (1) Meets the clinical diagnostic criteria for coronary heart disease; (2) No other organ system dysfunction

Exclusion criteria: (1) Patients who also suffer from other heart diseases; (2) Patients with pulmonary hypertension or heart failure; (3) Patients who are unable to receive specific treatment or follow-up.

The final included patients all signed informed consent forms. Randomly divide patients who meet the inclusion criteria into two groups: the control group and the experimental group. There are 30 patients in each group. The 60 coronary heart disease patients selected in the study can represent the entire patient population in the field of congenital heart disease diagnosis and treatment. The research results have external validity and can be extended to a wider range of scenarios.

Control group: Conventional two-dimensional ultrasound and surgery are used for diagnosis and treatment. Before conducting echocardiography on patients, it is necessary to inform them of the examination items and procedures. The patient takes off their upper body clothes and accessories and lies on the examination bed, maintaining a quiet state.

Experimental group: Three-dimensional echocardiography and image guided surgery were used for diagnosis and treatment. When performing echocardiography, doctors need to use appropriate gel to fit the Doppler probe with the patient’s chest for detection.

All patients underwent selective left and right coronary angiography via the grid artery or femoral artery, and underwent multi body projection coronary angiography according to Judkin's method. The diagnosis was independently made by two interventional cardiologists. If there are different results, the analysis can be independently conducted by the third senior physician, and the interventional cardiologist will use the self method for other patient data. The evaluation of coronary artery disease adopts an improved Gensimni scoring system: among the 8 main segments of the coronary artery, the most severe stenosis in each segment can be included in the scoring system, and the degree of coronary artery stenosis in each segment can be measured. The standard for stent implantation is that any stenosis in the left main coronary artery is ≥ 50% or in other coronary arteries is > 70%. This article divides the main blood vessels of the heart into: left main trunk, anterior descending branch, circumflex branch, and right coronary artery. According to the number of coronary artery lesions, they are divided into single vessel lesions, double vessel lesions, and multi vessel lesions. This article uses SPSS version 21.0 statistical software for data processing. All statistical data are subjected to normality tests and homogeneity of variance tests. Quantitative data is represented as mean ± standard deviation (xs), paired tests are used for comparison, frequency and percentage (%) are used for count data, and chi square tests are used for comparison.

CHD is generally divided into ventricular septal defect (Type 1), patent ductus arteriosus (Type 2), and atrial septal defect (Type 3). The basic information of the two groups of patients is shown in Table 3:

**Table 3:** Basic information of two groups of patients

Basic information	Index	Control group	Experimental group
Gender	Male	19	18
	Female	11	12
Age	3–4 years old	14	15
	5-6 years old	16	15
Disease type	Type 1	8	10
	Type 2	9	9
	Type 3	13	11

As shown in Table 3, there were 19 males and 11 females in the control group, and 18 males and 12 females in the experimental group. There were 8, 9, and 13 patients of Type 1, Type 2, and Type 3 in the control group, and 10, 9, and 11 patients of Type 1, Type 2, and Type 3 in the experimental group, respectively. The difference between the two groups of patients was not significant and was comparable.

### Comparison of diagnostic effects

The control group used traditional two-dimensional ultrasound for diagnosis of coronary heart disease. Traditional two-dimensional ultrasound has certain applications in the diagnosis of CHD. However, due to the fact that two-dimensional ultrasound is based on planar imaging technology, it is difficult to provide comprehensive and clear images of complex heart structures, which limits its accuracy and precision in clinical practice.

The experimental group used a combination of three-dimensional echocardiography and image-guided surgery for diagnosis. Three-dimensional echocardiography can use rotational scanning technology to obtain a true three-dimensional image of the heart. The combination of three-dimensional echocardiography and image guided surgery can provide more comprehensive and accurate diagnostic information, comprehensively observe and evaluate the structure, morphology, and functional status of the heart, and improve the diagnostic accuracy of CHD. Clear real-time 3D images help the cardiac team obtain the necessary information to determine whether a patient meets the criteria for transcatheter aortic valve repair surgery, and aid in planning and providing stunning views during surgery.

**Fig. 4:**
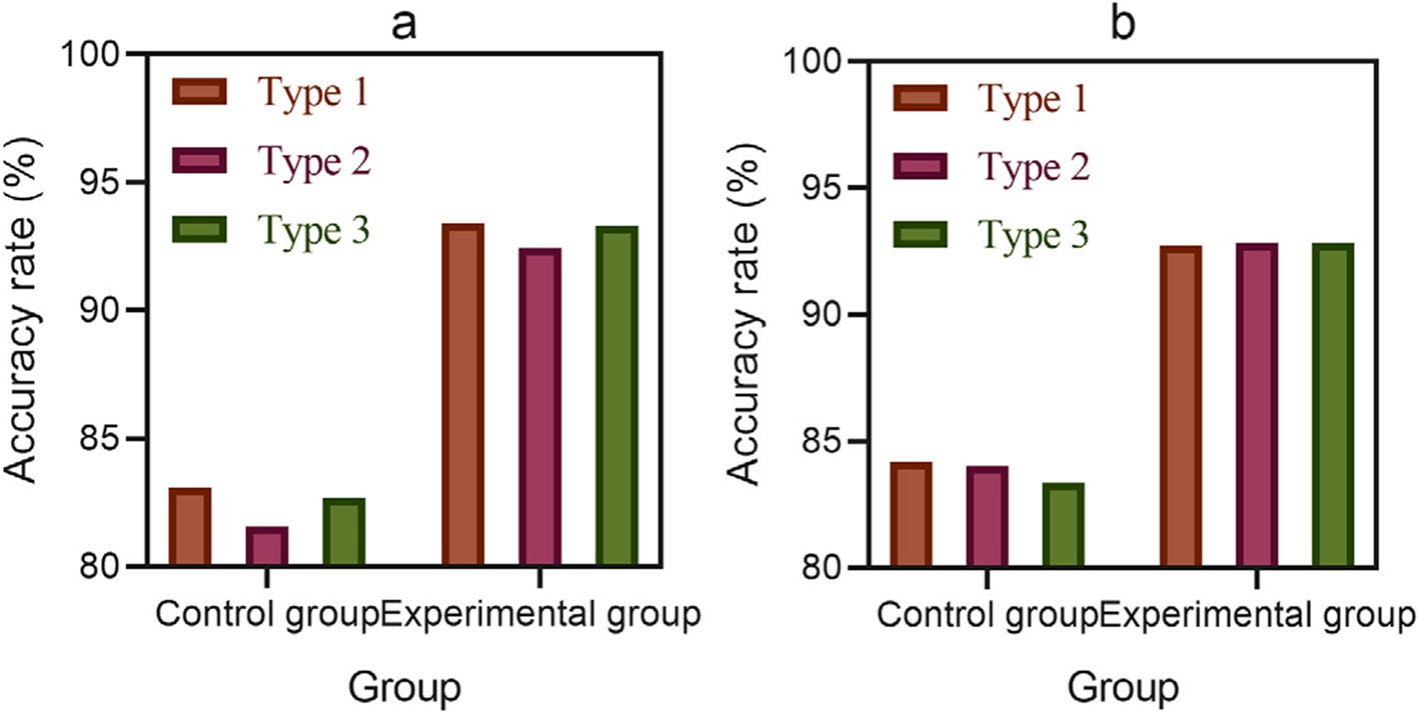
Accuracy of two different types of diagnosis in two diagnoses. **a** Accuracy of the first diagnosis for two different types of diagnoses. **b** The accuracy of the second diagnosis for two different types of diagnoses

Diagnosis of ventricular septal defect (Type 1), patent ductus arteriosus (Type 2), and atrial septal defect (Type 3) was performed on patients in the control and experimental groups. In order to reduce experimental error, two repeated diagnoses were performed, and the accuracy comparison is shown in Fig. 4 (the abscissa of Fig. 4 represents the control and experimental groups, and the ordinate represents the accuracy):

As shown in Fig. 4: In the first diagnosis in Fig. 4(a), the diagnostic accuracy rates for Type 1, Type 2, and Type 3 in the control group were 83.09%, 81.58%, and 82.70%, respectively; the diagnostic accuracy rates for Type 1, Type 2, and Type 3 in the experimental group were 93.40%, 92.45%, and 93.33%, respectively.

In the case of the second diagnosis in Fig. 4(b), the diagnostic accuracy rates for Type 1, Type 2, and Type 3 in the control group were 84.21%, 84.02%, and 83.38%, respectively; the diagnostic accuracy rates for Type 1, Type 2, and Type 3 in the experimental group were 92.73%, 92.82%, and 92.83%, respectively. From the two diagnostic results in Fig. 4, it can be seen that the diagnostic accuracy of the control group for different types of coronary heart disease is below 90%, while the diagnostic accuracy of the experimental group based on the combination of three-dimensional echocardiography and image-guided surgery is above 90%. This indicates that combining three-dimensional echocardiography with image-guided surgery can provide a more accurate and objective diagnosis of coronary heart disease.

### Comparison of treatment effects

#### Comparison of surgical risks

Image guided surgery can enable doctors to clearly see important tissues and structures around them during surgery through imaging technology, thus avoiding damage to important tissues and organs around them and reducing the risk of surgery.

The incidence of intraoperative risks in the control group and experimental group is shown in Table 4:

**Table 4:** Intraoperative risks (multiple choices)

Risk	Control group	Experimental group
Arrhythmia	5	1
cardiac arrest	3	0
Anesthesia accident	2	0
Complications of extracorporeal circulation	4	1

As shown in Table 4, there were 5 patients with arrhythmia in the control group and 1 patient in the experimental group, respectively; there were 3 and 0 patients with cardiac arrest, respectively; there were 2 and 0 patients with anesthesia accidents, respectively; there were 4 and 1 patients with complications of extracorporeal circulation, respectively. From Table 4, it can be seen that the control group treated with conventional surgery had a higher risk of arrhythmia, cardiac arrest, anesthesia accidents, and extracorporeal circulation complications, while the experimental group had a relatively lower risk incidence rate.

Three-dimensional echocardiography can provide real-time and dynamic cardiac image information, and image guided surgery can provide more accurate surgical positioning and operational information, thus enabling doctors to more accurately remove lesions during surgery, thereby avoiding accidental injury to surrounding tissues and organs and reducing surgical risks.

#### Comparison of surgical time

Image guided surgery can provide high-quality anatomical structure information. By using advanced medical imaging techniques such as three-dimensional echocardiography, doctors can obtain detailed and accurate cardiac structure information, including ventricular size and valve abnormalities, before surgery. This information can be imported into an image guided surgical navigation system to help doctors accurately locate and evaluate the location, size, and shape of lesions. By understanding the specific situation of the lesion in advance, doctors can carry out more precise surgical planning and design, thereby shortening the time during surgery.

**Fig. 5:**
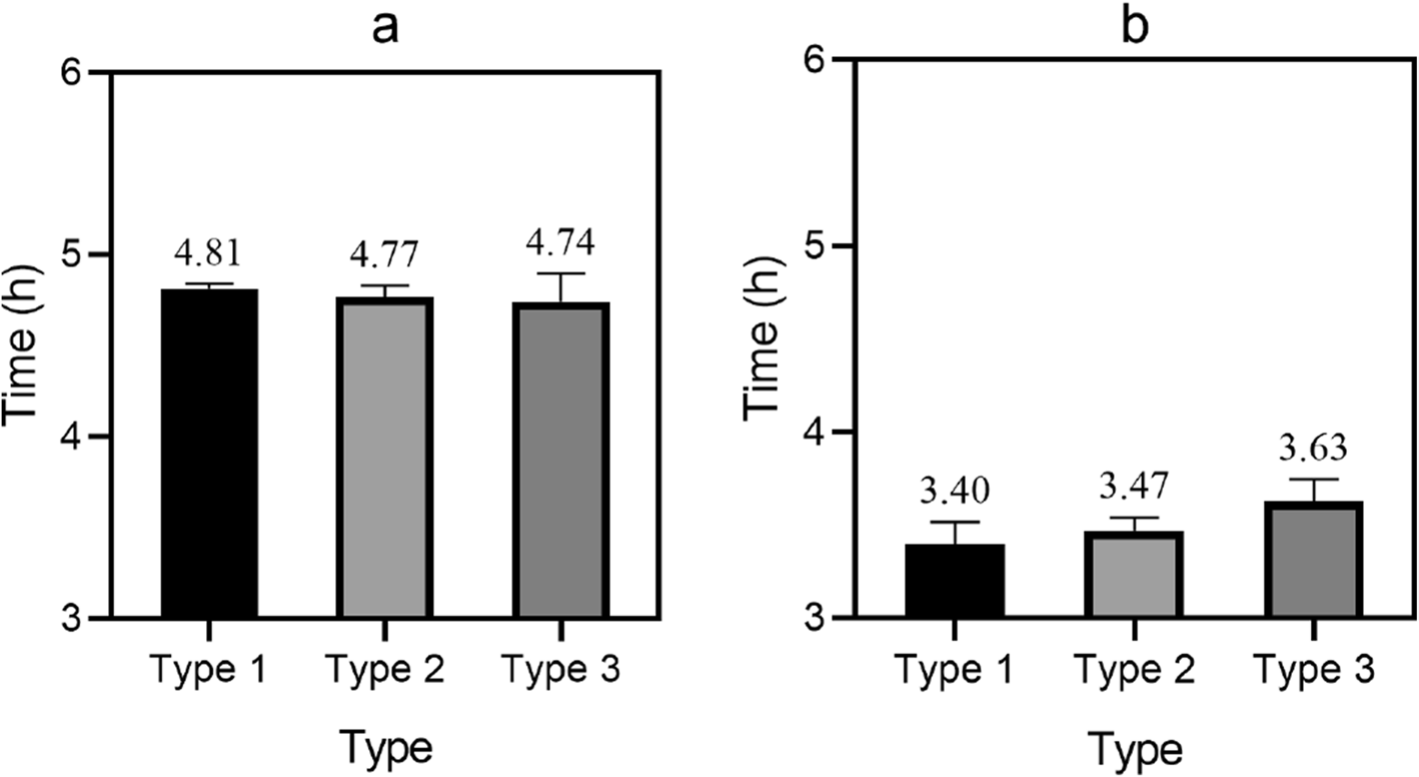
Average surgical time for two different types of patients; **a** Average surgical time of different types in the control group; **b** Average surgical time of different types in the experimental group

The surgery for CHD takes approximately 2 to 6 h. The condition of this disease is relatively complex and severe, and the surgical procedure is relatively difficult, so the entire surgical time is relatively long. The surgical time of patients of different genders was recorded, and the average surgical time of two groups of different types is shown in Fig. 5 (the horizontal axis of Fig. 5 represents Type 1, Type 2, and Type 3, and the vertical axis represents time):

As shown in Fig. 5: In Fig. 5(a), the average surgical time for different gender Types 1, 2, and 3 in the control group was 4.81 h, 4.77 h, and 4.74 h.

Figure 5(b) shows that the average surgical time for different gender Types 1, 2, and 3 in the experimental group was 3.40 h, 3.47 h, and 3.63 h. From the comparison results of surgical time in Fig. 5, it can be seen that the experimental group had a lower average surgical time for other types of coronary heart disease, with an average surgical time of less than 4 h, while the control group spent more time using conventional surgery, with an average surgical time of over 4 h.

Image guided surgery can provide real-time visual guidance. During the surgical process, traditional Chinese medicine doctors can obtain high-resolution image guidance in real time through image guidance. Real time image guidance can help doctors accurately locate and operate, avoid risks such as accidental cuts and injuries, and reduce surgical time. Doctors can perform precise manipulation based on real-time image guidance to improve surgical efficiency and success rate. Image guided surgery can provide a visual surgical plan. By combining image data such as three-dimensional echocardiography with surgical planning software, doctors can conduct simulation operations and training in a virtual surgical environment. Doctors can plan and optimize surgical procedures in advance, select the best surgical instruments and implants, and determine the surgical path and sequence of operations. This visual surgical plan can help doctors accurately grasp the key points of surgery, thereby shortening time and reducing unnecessary operations during surgery.

#### Comparison of incidence of complications

Image guided surgery can provide personalized treatment plans, and each patient’s CHD situation varies, requiring customized treatment plans. Through advanced imaging technology and three-dimensional reconstruction, personalized heart models can be generated for patients, and surgical planning and design can be carried out based on specific lesion characteristics. Doctors can pre simulate surgical operations in a virtual surgical environment and conduct simulation training to prevent and solve potential problems in advance. This personalized treatment plan can improve surgical effectiveness, reduce surgical time and incidence of complications. The combination of three-dimensional echocardiography and image guided surgery can achieve precise treatment for cardiac structural and functional abnormalities, maximize the protection of normal tissues, shorten surgery and recovery time, and improve treatment effectiveness.

**Fig. 6:**
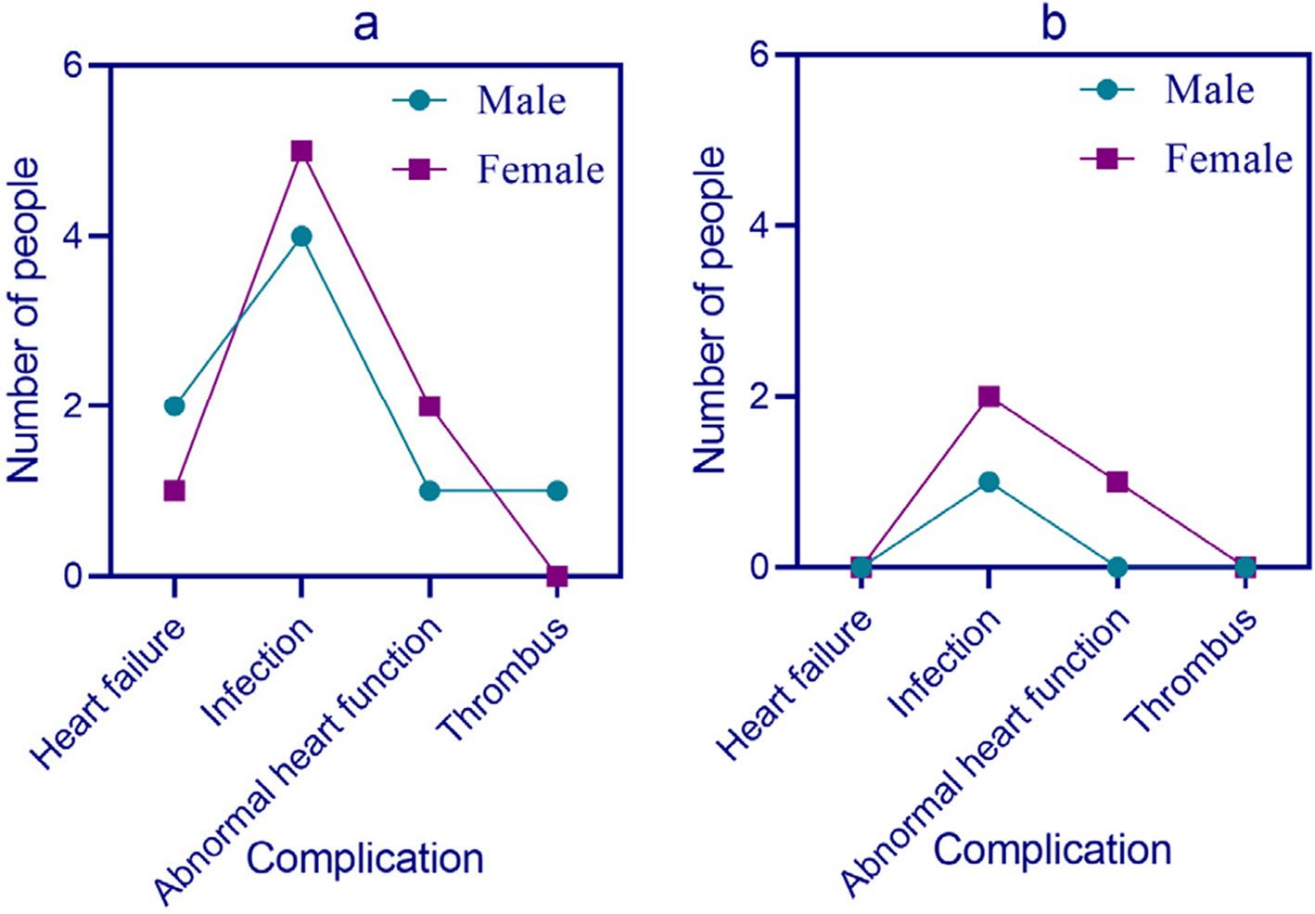
Postoperative complications in two groups of patients; **a** Postoperative complications in control group patients; **b** Postoperative complications in experimental group patients

Postoperative complications include heart failure, infection, cardiac dysfunction, and thrombosis. The incidence of postoperative complications in both groups of patients is shown in Fig. 6 (the x-axis of Fig. 6 represents the complications, and the y-axis represents the number of people):

As shown in Fig. 6: In Fig. 6 (a), there were 2 males and 1 female in the control group who experienced postoperative heart failure, respectively. There were 4 males and 5 females infected, respectively. There were 1 male and 0 female cases of thrombosis, respectively.

Figure 6 (b) showed that there were 0 males and 0 females in the experimental group who developed heart failure after surgery. The number of infected males and females was 1 and 2, respectively. There were 0 males and 0 females who developed blood clots, respectively. From the postoperative complications results in Fig. 6, it can be seen that the control group based on conventional surgical treatment had a higher number of patients with postoperative complications, while the experimental group based on a combination of three-dimensional echocardiography and image-guided surgery had a lower number of patients with postoperative complications.

This article used a combination of image guided surgery and three-dimensional echocardiography to diagnose and treat CHD, and evaluated the treatment effect. The experimental results showed that the combined use of three-dimensional echocardiography and image guided surgery significantly outperformed traditional treatment methods.

#### Comparison of physical conditions

**Fig. 7:**
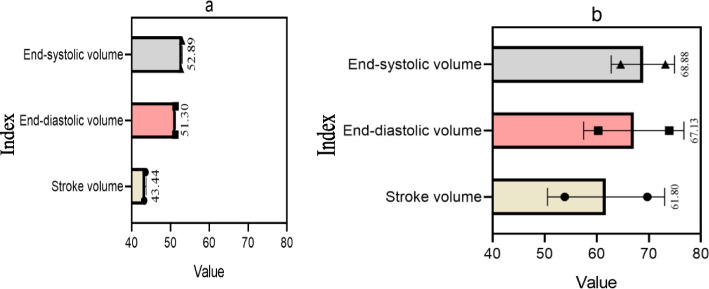
Physical data of two groups of patients before and after surgery; **a** Body data of two groups of patients before surgery; **b** Postoperative body data of two groups of patients

The stroke output, end-diastolic volume, and end-systolic volume were recorded in both groups before and after surgery. The range of stroke output per normal person was 60–80 ml. The normal value of left ventricular end diastolic volume was 75–160 ml, and the end systolic volume was about 75 ml. The stroke output, end diastolic volume, and end systolic volume of the two groups before and after surgery are shown in Fig. 7 (the abscissa of Fig. 7 represents values, and the ordinate represents different indicators):

As shown in Fig. 7: in Fig. 7 (a), the stroke output, end diastolic volume, and end systolic volume of the control group before surgery were 43.25 ml, 51.31 ml, and 52.76 ml, respectively; the stroke output, end diastolic volume, and end systolic volume of the experimental group were 43.63 ml, 51.29 ml, and 53.01 ml, respectively; the average stroke output of the two groups was 43.44 ml.

Figure 7 (b) showed that the stroke output, end diastolic volume, and end systolic volume of the control group after surgery were 53.87 ml, 60.33 ml, and 64.59 ml, respectively; the stroke output, end diastolic volume, and end systolic volume of the experimental group were 69.72 ml, 73.93 ml, and 73.17 ml, respectively; the average stroke output of the two groups was 61.80 ml.

## Discussion

The combination of image guided surgery and three-dimensional echocardiography could bring greater advantages in the diagnosis and treatment of CHD. Specifically manifested in the following aspects:

The data in Fig. 4 indicated that the diagnostic accuracy of the experimental group was relatively high, indicating that three-dimensional echocardiography had better accuracy in the diagnosis of CHD, which helped to improve the diagnostic accuracy and treatment effectiveness of patients.

Due to the complex and diverse cardiac structure of CHD, two-dimensional ultrasound may be limited by the limitations of the image in the diagnosis process, making it difficult to accurately determine the location, degree, and shape of the lesion. Three-dimensional echocardiography can provide more angle and perspective information, helping doctors comprehensively understand the condition of the lesion, especially for complex lesions such as valve defects and ventricular septal defects. Three dimensional echocardiography can provide more accurate diagnosis and evaluation. Three-dimensional echocardiography can also provide more accurate measurement of heart size. Traditional two-dimensional ultrasound can only measure the size of the heart cavity and valve through two-dimensional sections, which may have geometric assumptions and measurement errors. Three-dimensional echocardiography can provide more accurate measurements of heart size and volume, helping doctors better evaluate the condition and formulate treatment plans.

Table 4 showed that the experimental group had a lower surgical risk, and image guided surgery could help improve surgical safety and success rate. Three-dimensional echocardiography provided real-time visualization information for many operations, which could guide doctors during surgery, avoid damage to important surrounding tissues and organs, and reduce surgical risks. Image guided surgery could provide more accurate positioning information, enabling doctors to more accurately remove lesions, reduce the scope of resection, and maximize the preservation of normal tissue.

The data in Fig. 6 showed that the number of postoperative complications in the experimental group was lower than that in the control group. The combined application of image guided surgery and three-dimensional echocardiography could provide doctors with accurate, detailed, and visualized cardiac structural information, and help them navigate and operate in real-time during surgery. By using high-resolution medical imaging technologies such as three-dimensional echocardiography, medical students could obtain more detailed and accurate cardiac structural information before surgery. This information could be imported into image guided surgery to help doctors accurately locate and evaluate the location, size, and shape of lesions. Compared to traditional surgery, image guided surgery could better guide doctors in surgical operations and reduce postoperative complications.

The combined application of image guided surgery and three-dimensional echocardiography is not only more convenient and accurate for doctors, but also provides patients with a better treatment experience and effect. With the continuous progress of technology, the combination of image guided surgery and three-dimensional echocardiography would play an increasingly important role in the treatment of CHD.

## Conclusions

CHD is a common heart disease that requires a series of methods for its diagnosis and treatment. This article improves the diagnosis and treatment of CHD by combining three-dimensional echocardiography and image guided surgery, and improves the level and quality of medical services. The combination of three-dimensional echocardiography and image guided surgery plays an important role in the diagnosis and treatment of CHD. The experimental results indicated that the combination of three-dimensional echocardiography and image guided surgery had higher diagnostic accuracy and lower surgical risk, and the diagnostic and therapeutic effects were significantly better than traditional diagnostic methods and surgeries. The combination of image guided surgery and three-dimensional echocardiography could bring more advantages, improve the diagnosis and treatment effect of CHD, and provide patients with a better treatment experience. It could also provide surgeons with a better operating experience. This method is expected to be widely promoted and applied in clinical applications. The research results of this article can provide scientific basis for clinical application and provide better training plans for clinical practice. However, the sample size chosen for this study is not large enough and lacks a certain degree of reliability. With the continuous development of medical technology, three-dimensional echocardiography and image guided surgery would be increasingly widely used in the diagnosis and treatment of heart disease, bringing more accurate and effective treatment to patients.

## Data Availability

All data generated or analysed during this study are included in this published article.

## References

[1] Li X, Sui J, Wang Y. Three-Dimensional Reconstruction of Fuzzy Medical Images Using Quantum Algorithm. IEEE Access. 2020;8:218279–88.

[2] Kinjal D, Rabinowitz EJ, Epstein S. Physiologic diagnosis of congenital heart disease in cyanotic neonates. Curr Opin Pediatr. 2019;31(2):274–83. 30730315 10.1097/MOP.0000000000000742

[3] Rima A, Curran L, Zhao Y, Levine JC, Chinn E, Moon-Grady AJ. An ensemble of neural networks provides expert-level prenatal detection of complex congenital heart disease. Nat Med. 2021;27(5):882–91. 33990806 10.1038/s41591-021-01342-5 PMC8380434

[4] Letourneau Karen M., Horne D, Soni RN, McDonald Keith R., Fransoo RR. “Advancing Prenatal Detection of Congenital Heart Disease: A Novel Screening Protocol Improves Early Diagnosis of Complex Congenital Heart Disease.” J Ultrasound Med. 2018;37(5):1073–9. 29027708 10.1002/jum.14453

[5] George M, Shum K, Gupta T, Chakravorty S, Kachur S, Bienvenu L, et al. Echocardiography in congenital heart disease. Prog Cardiovasc Dis. 2018;61(5–6):468–75. 30445162 10.1016/j.pcad.2018.11.004

[6] Di Salvo Giovanni, Miller Owen, Narayan Sonya Babu, Lei Wei, Budts Werner, Valsangiacomo Buechel Emanuela R, et al. “Imaging the adult with congenital heart disease: a multimodality imaging approach—position paper from the EACVI.” Eur Heart J Cardiovasc Imaging. 2018;19(10):1077–98. 30084968 10.1093/ehjci/jey102

[7] Medvedofsky Diego, Maffessanti Francesco, Weinert Lynn, Tehrani David M., Narang Akhil, Addetia Karima, et al. “2D and 3D echocardiography-derived indices of left ventricular function and shape: relationship with mortality.” JACC: Cardiovasc Imaging. 2018;11(11):1569–79. 29153577 10.1016/j.jcmg.2017.08.023 PMC5945352

[8] Chinh ND, Ha LM, Sun G, Anh LQ. "Short time cardio-vascular pulses estimation for dengue fever screening via continuous-wave Doppler radar using empirical mode decomposition and continuous wavelet transform." Biomed Signal Process Control. 2021;65:102361.

[9] Huy Tran Quang, Huynh Huu Tue, Ton That Long, Tran Duc-Tan. “Deterministic compressive sampling for high-quality image reconstruction of ultrasound tomography.” BMC Med Imaging. 2017;17(1):34. 28545406 10.1186/s12880-017-0206-8 PMC5445364

[10] Brida M, Gatzoulis MA. Adult congenital heart disease: past, present and future. Acta Paediatr. 2019;108(10):1757–64. 31254360 10.1111/apa.14921

[11] Mone Fionnuala, Eberhardt R. Y., Morris R. K., Hurles M. E., McMullan D. J., Maher E. R., et al. “COngenital heart disease and the Diagnostic yield with Exome sequencing (CODE) study: prospective cohort study and systematic review.” Ultrasound Obstet Gynecol. 2021;57(1):43–51. 32388881 10.1002/uog.22072

[12] Vladimirovna SV, Vladimirovna ME, Singh S, Bugalia A. Pregnancy with congenital heart disease. Science and innovation. 2023;2(D4):127–36.

[13] Vaidya Anand, Flores Shahida K., Cheng Zi-Ming, Nicolas Marlo, Dahia Patricia L.M. “EPAS1 mutations and paragangliomas in cyanotic congenital heart disease.” N Engl J Med. 2018;378(13):1259–61. 29601261 10.1056/NEJMc1716652 PMC5972530

[14] Ahmed MR, Ashrafi AF, Ahmed RU, et al. DoubleU-NetPlus: a novel attention and context-guided dual U-Net with multi-scale residual feature fusion network for semantic segmentation of medical images. Neural Comput & Applic. 2023;35:14379–401.

[15] Lambert James, Mariana Lamacie, Babitha Thampinathan, Mustafa A Altaha, Maryam Esmaeilzadeh, Mark Nolan, et al. “Variability in echocardiography and MRI for detection of cancer therapy cardiotoxicity.” Heart. 2020;106(11):817–23. 32098808 10.1136/heartjnl-2019-316297

[16] Yang B, Liu M, Wang Y, Zhang K, Meijering E. Structure-Guided Segmentation for 3D Neuron Reconstruction. IEEE Trans Med Imaging. 2022;41(4):903–14. 34748483 10.1109/TMI.2021.3125777

[17] Lytzen R, Potiny P, Rigdon J, Morello M, Tcheandjieu C, Romfh A, et al. "Live-born major congenital heart disease in Denmark: incidence, detection rate, and termination of pregnancy rate from 1996 to 2013." JAMA Cardiol. 2018;3(9):829–37. 10.1001/jamacardio.2018.2009 PMC6233653 30027209

[18] Chandramohan Dhasarathan, Shanmugam M, Manish Kumar, Diwakar Tripathi, Shailesh Khapre, Achyut Shankar. A nomadic multi-agent based privacy metrics for e-health care: a deep learning approach. Multim Tools Appl. 2024;83(3):7249–72. 10.1007/s11042-023-15363-4 PMC10241612 37362729

[19] Muraru Denisa, Luigi P Badano, Yasufumi Nagata, Elena Surkova, Yosuke Nabeshima, Davide Genovese, et al. “Development and prognostic validation of partition values to grade right ventricular dysfunction severity using 3D echocardiography.” Eur Heart J Cardiovasc Imaging. 2020;21(1):10–21. 31539046 10.1093/ehjci/jez233

[20] Gandhi Sumeet, Wassim Mosleh, Joshua Shen, Chi-Ming Chow. “Automation, machine learning, and artificial intelligence in echocardiography: a brave new world.” Echocardiography. 2018;35(9):1402–18. 29974498 10.1111/echo.14086

[21] Lang Roberto M, Addetia Karima, Narang Akhil, Mor-Avi Victor. 3-Dimensional echocardiography: latest developments and future directions. JACC Cardiovasc Imaging. 2018;11(12):1854–78. 30522687 10.1016/j.jcmg.2018.06.024

